# Fungicides Reduce the Abundance of Yeast-like Symbionts and Survival of White-Backed Planthopper *Sogatella furcifera* (Homoptera: Delphacidae)

**DOI:** 10.3390/insects11040209

**Published:** 2020-03-27

**Authors:** Kun Pang, Shengzhang Dong, Peiying Hao, Tongtong Chen, Xinlong Wang, Xiaoping Yu, Huafeng Lin

**Affiliations:** 1 School of Plant Protection, Anhui Agricultural University, Hefei 230036, China; pangk@cjlu.edu.cn; 2 W. Harry Feinstone Department of Molecular Microbiology and Immunology, Bloomberg School of Public Health, Johns Hopkins University, Baltimore, MD 21205, USA; sdong13@jhu.edu; 3 Zhejiang Provincial Key Laboratory of Biometrology and Inspection & Quarantine, College of Life Sciences, China Jiliang University, Hangzhou 310018, China; haopy@cjlu.edu.cn (P.H.); Chen15355470398@163.com (T.C.); wangxinlong166@163.com (X.W.)

**Keywords:** YLS diversity, DGGE, *Ascomycetes* symbiotes, effective strategy, synergistic effects

## Abstract

The white-backed planthopper (WBPH) *Sogatella furcifera* is one of the most harmful pests of rice in Southeast Asia. The fat body of WBPH harbors intracellular yeast-like symbionts (YLS). YLS are vertically transmitted to WBPH offspring by transovarial infection. YLS play an important role in the WBPH life cycle. YLS diversity and function have been extensively studied in the brown planthopper (BPH) and small brown planthopper but not in WBPH, even though a novel strategy for controlling the BPH based on suppressing YLS has been proposed. Here, using denaturing gradient gel electrophoresis, we identified 12 unique fungal sequences among YLS of WBPH, and five of them represented uncultured fungi. We then fed WBPH with rice plants treated with different fungicides [70% propineb wettable powder (WP) (PR), 70% propamocarb hydrochloride aqueous solution (AS) (PH), 25% trifloxystrobin and 50% tebuconazole water-dispersible granules (WG) (TT), 40% pyrimethanil suspension concentrate (SC) (PY), and 50% iprodione SC (IP)] and evaluated their effects on YLS abundance and WBPH survival rate. Both YLS abundance and adult WBPH survival rate were significantly decreased upon feeding fungicide-treated rice plants, and exposure to 50% IP resulted in the strongest reduction. The abundance of two Sf-YLS species (*Ascomycetes* symbiotes and *Cla*-like symbiotes) was significantly reduced upon exposure to 50% IP. The counts of *Ascomycetes* symbiotes, the most abundant YLS species, were also suppressed by the other fungicides tested. In conclusion, 50% IP was the most effective fungicide, reducing YLS abundance and WBPH survival rate under controlled conditions, suggesting its potential use to control WBPH.

## 1. Introduction

The white-backed planthopper (WBPH) *Sogatella furcifera* (Horváth) is one of the most harmful pests in paddy fields in southeast Asia [1,2]. Similar to other rice planthoppers, the WBPH harbors intracellular yeast-like symbionts (YLS) in the fat body [3]. YLS occur not only in planthoppers but also in some aphids, scale insects, leafhoppers and cicadas [4,5,6]. The diversity of YLS in rice planthoppers has been recently demonstrated [7,8,9], and has been found to be altered in response to insecticides and environmental factors [10,11]. The first YLS species identified in the brown planthopper (BPH), by using 18S rDNA sequencing, belongs to the class Pyrenomycetes [12,13,14]. Subsequent parsimony analysis placed this YLS species within *Cordyceps* (Euascomycetes: Hypocreales: Clavicipitaceae), a genus of filamentous entomopathogenic ascomycetes [15]. Dong et al. [8] identified two other YLS species, *Pichia*-like and *Cryp*-like symbionts of BPH, using nested polymerase chain reaction (PCR) and in situ hybridization. By using denaturing gradient gel electrophoresis (DGGE), several other YLS species, such as species of *Saccharomycetales* and *Debaryomyces hansenii*, were identified [7]. Cao et al. [9] successfully isolated a new YLS species (*Pichia anomala*) from the small brown planthopper (SBPH) by in vitro culture.

YLS play various roles in the brown planthopper (BPH), e.g., amino acid biosynthesis, steroid synthesis, and nitrogen recycling [16,17]. For example, BPH and the associated YLS have developed a mutualistic system for steroid biosynthesis, in which YLS produce ergosta-5,7,24(28)-trienol, which is utilized for cholesterol synthesis by the host [18]. In addition, YLS provide essential amino acids that the BPH is unable to synthesize and also they can use uric acid, a nitrogenous waste stored in the BPH body, for nitrogen recycling [17,19]. These functions can enable the BPH to exist, feeding solely on rice phloem sap, which is nutritionally imbalanced. Furthermore, YLS are intimately associated with each developmental stage of the host [7,20]. 

The current control of the WBPH primarily relies on the use of synthetic insecticides, leading to pesticide resistance in WBPHs in most areas. For example, the WBPH has developed moderate resistance to buprofezin, and approximately 32% of WBPH field populations exhibit resistance to imidacloprid in eastern China [21]. A novel control strategy based on inhibiting YLS growth was recently proposed, considering the essential function of YLS in the BPH. Several fungicides that effectively inhibited YLS also killed BPHs [22,23]. To test whether this control strategy could also be applied to WBPHs, we used denaturing gradient gel electrophoresis (DGGE) to investigate the composition of WBPH YLS. We then investigated the abundance of two YLS species and WBPH survival in response to five fungicides that are often used to control plant pathogens [24,25,26,27,28]. These findings may guide the development of an effective management strategy against WBPH.

## 2. Materials and Methods

### 2.1. Insects and Fungicides

The WBPH populations were collected from paddy fields in Hangzhou (E120°12, N30°16), Zhejiang, China. The insects were reared using rice plants of the susceptible variety Taichung Native 1 (TN1). Insects were kept in a psychrometric room at 26 ± 1 °C, with a relative humidity of 70%–80% and a 16 h light/8 h dark photoperiod. The five fungicides tested were 70% propineb wettable powder (WP) (PR), 70% propamocarb hydrochloride aqueous solution (AS) (PH), 25% trifloxystrobin and 50% tebuconazole water-dispersible granules (WG) (TT), 40% pyrimethanil SC (PY), and 50% iprodione suspension concentrate (SC) (IP). The PR is protectant fungicide. PH, TT PY, and IP are systemic fungicides. The fungicides were all from Bayer Crop Science Hangzhou China Co., Ltd. All fungicides were dissolved in deionized water purified with Milli-Q^®^ Type 1 Ultrapure Water Systems (Millipore, Billerica, MA, USA).

### 2.2. Nested PCR, DGGE, and Sequence-based Analysis of YLS Diversity in WBPH

Nested PCR consisted of two rounds of PCR. The first-round PCR was performed using the universal fungal primers NS5–ITS4, and the second-round PCR was performed using GCclampITS1–ITS2 and GCclampITS3–ITS4 primers (Table 1) [7,29,30]. The DNA template for first-round PCR was extracted from YLS. The YLS were purified from the abdomen of WBPH using a method described by Noda and Omura [31]. YLS genomic DNA was extracted using a Yeast DNA Mini Kit (Tiangen, Beijing, China), which is a spin-column purification method based on the enzymolysis of yeast cells with recombinant Lyticase enzymes (Sigma, St. Louis, MO, USA). Diluted amplicons from the first-round PCR were used as the template for second-round PCR. The PCR was performed as described by Shentu et al. [22]. The thermal cycles were as follows: 95 °C for 4 min, followed by 35 cycles of 95 °C for 30 s, 57 °C for 30 s and 72 °C for 45 s, and a final extension of 72 °C for 10 min. DGGE was performed using a Protean II system (Bio-Rad, Hercules, CA, USA), and the DGGE protocol and working procedure were as described by Hou et al. [7]. The PCR products were loaded directly onto an 8.0% (wt/v) polyacrylamide gels in 1× TAE. The 8.0% (wt/v) polyacrylamide gel was made by denaturant stock solution (40% acrylamide/bis, 50×TAE buffer, and urea) ranging from 20% to 55%. Electrophoresis was performed for 12.5 h at a constant voltage of 80 V and a temperature of 60 °C. After electrophoresis, the DNA in the DGGE gel was detected by a routine silver-staining protocol [32], and then photographed with a video system (Bio Image Products, Ann Arbor, MI, USA).

The DGGE gels were silver-stained. Dark-stained DGGE bands were excised using a sterile blade and then incubated overnight at 4 °C in Tris–EDTA buffer (pH 8.0) to allow for DNA diffusion out of the polyacrylamide matrix. The eluate was then used directly in amplification reactions, as described above. PCR products were recovered, ligated into the pMD19-T easy vector, and used to transform competent cells. The cloned sequences were then sequenced by Sangon Biotech Co. (Shanghai, China), Ltd. and identified using nucleotide Basic Local Alignment Search Tool (BLAST) available at the National Center for Biotechnology Information [33]. The results with the highest degree of similarity/homology are presented in Table 2.

### 2.3. Foliar Spray of Rice Plants with Fungicides

The rice seedlings at the three-leaf stage were used for foliar spray, and the bud sheath and incomplete leaf were removed to expose the tender leaves to facilitate the absorption of the fungicide and the feeding of the planthopper. The removal was identical across all treatments. The stems were washed, getting rid of the excess water and dried by absorbent paper. The fungicides were dissolved in water according to the recommended concentrations in the field [22]. The fungicide was uniformly sprayed on the surface of stems and the stems were air-dried for 1 h. During the fungicide spraying, the rice seedlings were fully covered with the fungicides until droplets were uniformly formed on the rice seedlings. A quantitative sprayer (INGLEMIREPHARM’S, Hangzhou, Zhejiang, China) was used to minimize the difference between each experiment. After drying, three seedlings were added to each test tube. Thirty adult WBPH females emerged within 24h were placed in each test tube, fed for up to five days and the tubes were sealed with two layers of gauze. The deionized water was used as the control. The treatments (five fungicides and one control) were all repeated three times.

### 2.4. Quantification of YLS and WBPH Survival Rate

Adult WBPH females fed on the treated plants and control plants before were collected on days 1, 3, and 5 after application, then the whole insects were sterilized by 3 min immersions in 75% ethanol. Fat bodies were dissected on ice and collected in a sterile tube. Fat bodies were homogenized in 0.02 M phosphate-buffered saline (PBS) at pH 7.4. Percoll (Pharmacia, Sweden) was added to the homogenate to give a final concentration of 30%, and then centrifuged at 2000 ×g for 10 min. The pellet was suspended in PBS containing 250 mM sucrose and 75% Percoll, and centrifuged at 100,000 ×g for 20 min. The YLS were collected from the 65%–85% region of the Percoll gradient, the purity of YLS was assessed using a microscope and the number of YLS was calculated according to the formula stipulated by Chen et al. [34]. YLS cells in each sample were counted at least three times. The fat bodies collected from three WBPHs per sample were used to count the YLS number, and three replicates for each time point were performed. The number of surviving WBPH in the different treatment groups was recorded on days 1, 3, and 5, and the survival rate was calculated as a percentage of the original adults in the test tubes (Table 3).

### 2.5. Absolute Quantitative Real-time PCR Analysis of YLS Species

Adult WBPH females that had survived for 5 days in the treatment groups were collected, and YLS were isolated and quantified by quantitative PCR (qPCR). The 18S-ITS rDNA gene fragment of each YLS species was amplified by PCR. The PCR volume and procedure were as described by Shentu et al. [22]. The two primer pairs (as1f-as1r and ch1f-ch1r) used for PCR amplification were designed using Prime Premier 6.0, based on the partial sequence obtained from the WBPH YLS DGGE bands (Table 2). The resulting PCR products were extracted from 1.5% agarose gel, cloned into plasmid vector, sequenced, and compared with the corresponding sequence from the YLS DGGE band.

YLS abundance in WBPH was determined by qPCR using the respective primer sets (Table 1) [35]. Each DNA sample was analyzed three times. qPCR was performed in a 15 μL total reaction volume containing 7.5 μL of SYBR^®^ Premix Ex Taq™ (Takara, Dalian, China) (2×), 5 μL of template, 0.3 μL of forward primer, 0.3 μL of reverse primer, 0.3 μL of ROX Reference Dye (50×) and 1.6 μL of ddH_2_O. The qPCR reactions were 95 °C for 3 min, followed by 40 cycles of 95 °C for 30 s and 57 °C for 30 s.

### 2.6. Statistical Analysis

The YLS counts in different WBPH treatment groups were compared by using SPSS 18.0 (IBM, Armonk, New York, USA) and single-factor analysis of variance (one-way ANOVA). Statistical mean separations were performed by Tukey’s method; values of *p* < 0.05 were considered to indicate statistical significance.

## 3. Results

### 3.1. YLS Diversity in WBPH

**Figure 1:**
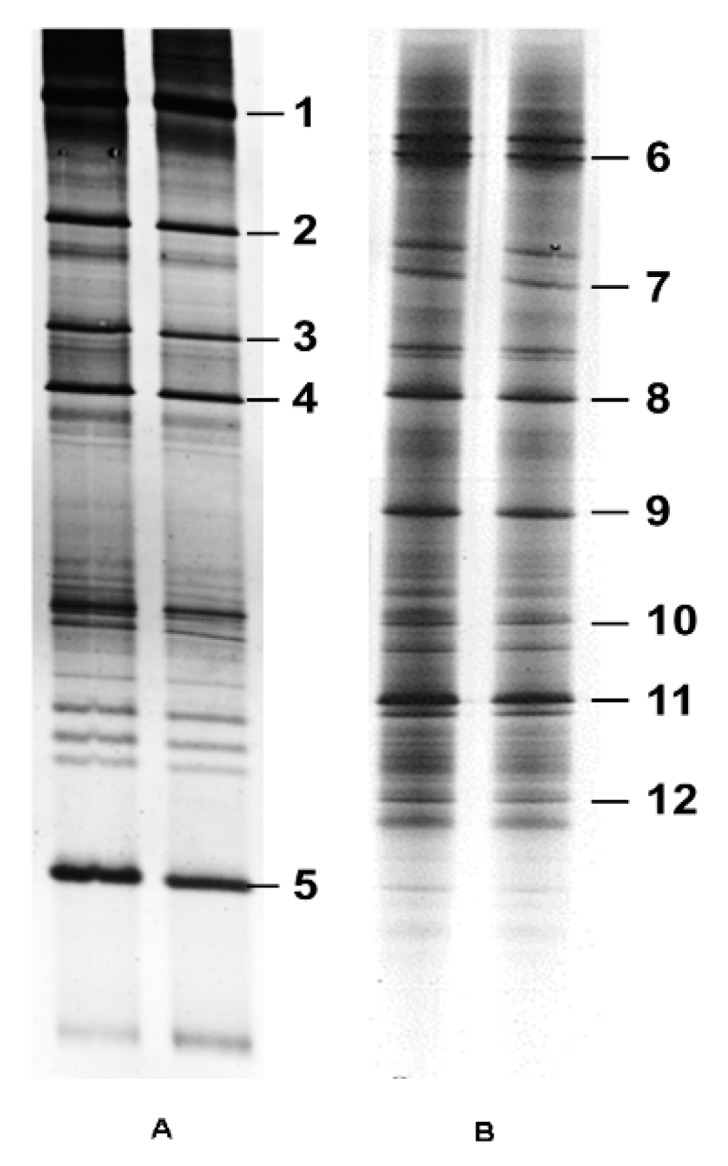
Fungal 18S-ITS fragments obtained from WBPH YLS by DGGE. (**A**) PCR-amplified fragment using the first-round primers NS5-ITS4 and second-round primers GCclampITS1-ITS2. (**B**) PCR-amplified fragment using the first-round primers NS5-ITS4 and second-round primers GCclamp ITS3-ITS4. The corresponding bands were sequenced.

Using the nested PCR-DGGE approach, 12 DGGE bands were identified and selected for further analysis based on their position and abundance in the gel. The bands were sequenced using the first-round primers NS5–ITS4 and second-round primers GCclampITS1–ITS2 or GCclampITS3–ITS4 (Figure 1). The size of the bands varied from 244 to 469 bp. Nucleotide BLAST analysis identified seven fungal species [*Alternaria alternata* isolate Alt-C71, *Periconia macrospinosa*, *Alternaria alternata* strain PB-56, *Fusarium* sp., *Cladosporium halotolerans*, *Naganishia albida*, and *S. furcifera* YLS (*Ascomycetes* symbiotes)] and five uncultured fungi (Table 2). With the exception of *S. furcifera* YLS (*Ascomycetes* symbiotes), which was previously identified in BPH by Noda et al. [14], all other YLS species were identified in WBPH for the first time.

### 3.2. Effect of Fungicides on YLS Abundance and WBPH Survival Rate

**Figure 2:**
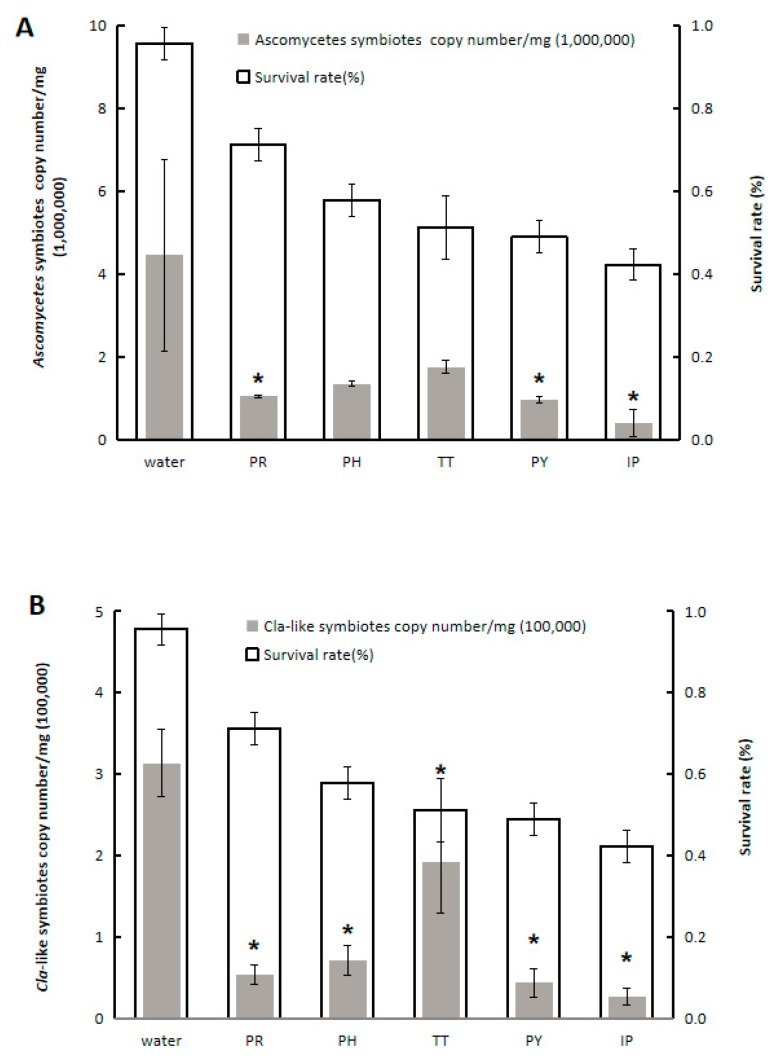
Effect of fungicides on the abundance of *Ascomycetes* symbiotes (**A**) and *Cla*-like symbiotes (**B**), and on survival rate of female adult WBPH. The abundance of *Ascomycetes* symbiotes and *Cla*-like symbiotes was determined by qPCR. On the X-axis PR, 70% propineb wettable powder (WP); PH, 70% propamocarb hydrochloride aqueous solution (AS); TT, 25% trifloxystrobin and 50% tebuconazole water-dispersible granules (WG); PY, 40% pyrimethanil suspension concentrate (SC); IP, 50% iprodione SC; The asterisks represents the significant differences (<0.01) which was tested by Tukey’s method.

The five tested fungicides reduced YLS numbers and WBPH survival rate at 5 days after exposure, where the IP treatment exerted the strongest effect (Figure 2 and Table 3). The YLS numbers in WBPH treated with IP decreased to 23.90% (*F* = 120.158, df = 5,12, *p* < 0.0001), 15.92% (*F* = 136.121, df = 5,12, *p* < 0.0001), and 9.18% (*F* = 116.348, df = 5,12, *p* < 0.0001) when compared to control group on days 1, 3, and 5, respectively. Furthermore, the survival rate of WBPH treated with IP declined from 93.33% to 42.22% at 5 days after female adult release.

Data were collected on days 1, 3, and 5 after the exposure of WBPH adults to rice plants treated with 70% propineb wettable powder (WP) (PR), 70% propamocarb hydrochloride aqueous solution (AS) (PH), 25% trifloxystrobin and 50% tebuconazole water-dispersible granules (WG) (TT), 40% pyrimethanil SC suspension concentrate (PY), and 50% iprodione suspension concentrate (SC) (IP). All the fungicides were dissolved in water according to the recommended concentration in the field [22]. Different letters denote significant differences in values among treatments in the same column and same day (*p* < 0.05). The ratio index represents that every treatment of YLS number is divided by the number of YLS in the control.

### 3.3. Effect of Fungicides on the Abundance of Two YLS Species

The effect of the five tested fungicides on the abundance of two YLS species (*Ascomycetes* symbiotes and *Cla*-like symbiotes) in WBPH was determined by qPCR (Figure 2). The abundance of *Ascomycetes* symbiotes significantly decreased upon IP, PY, or PR treatment (Figure 2); IP treatment exerted the greatest inhibitory effect (*F* = 6.825, df = 5,12, *p* = 0.003). Consistent with the observations for *Ascomycetes* symbiotes, IP treatment also resulted in the highest reduction in the abundance of *Cla*-like symbiotes. The inhibitory effect of IP on these two YLS species was the most significant, which was consistent with the results shown in Table 3. Notably, *Ascomycetes* symbiotes were the dominant species in WBPH, and they exhibited the greatest drop in abundance in response to the fungicide.

## 4. Discussion

Using a DGGE approach, we identified seven fungal species and five uncultured fungi associated with WBPHs that have caused tremendous losses to the rice in Asia. Furthermore, we showed that some fungicide treatments under the conditions of this study were detrimental to YLS, as well as to the WBPH host, with potential implications for developing an alternative strategy for controlling the WBPH in the field.

YLS play an important role in the entire life cycle of BPH [36,37]. However, the biological role of individual YLS species in the host has not been investigated. In the current study, we found that the impact of the YLS on WBPH survival depends on the YLS species. For example, reduced numbers of *Ascomycetes* symbiotes were associated with the lowest survival of WBPH, while *Cla*-like symbiotes only mildly affected the mortality of WBPH upon fungicide treatments. 

Several studies focusing on the inhibitory effect of fungicides on the diversity and abundance of BPH symbionts have been published [22,23]. For example, the fungicide 5% jinggangmycin AS strongly inhibits the endosymbionts from BPH [38]. In addition, six antibiotics were found to reduce the abundance of YLS and the BPH survival rate [34], and in another recent study, eight fungicides inhibited the abundance of YLS in BPH [22]. We also tested the inhibitory effect of five fungicides on two YLS species in WBPH. The survival rate of WBPH was greatly reduced with a decreasing YLS abundance. The findings of the current study suggest that fungicides that inhibit the growth of YLS could be employed synergistically with insecticides for improved suppression of WBPH populations, with the added potential benefit of minimizing the dose of insecticides used in paddy fields. Such an approach could represent a novel strategy to manage WBPH and other sap-sucking insects harboring fungal symbionts.

## 5. Conclusions

The current study explored the role of fungicides on suppressing YLS diversity and WBPH survival in white-back planthopper (WBPH). With a modified DGGE technique, we identified 12 unique fungal sequences in WBPH, and 11 of them were first reported. Five common fungicides were used to test their effects on survival rates of WBPH and YLS abundance, and 50% IP was the most effective fungicide to reduce the YLS abundance and adult WBPH survival. The abundance of two dominant *Sf*-YLS species (*Ascomycetes* symbiotes and *Cla*-like symbiotes) were significantly suppressed by all the tested fungicides, and 50% IP showed the highest inhibition on these two *Sf*-YLS species. Taken together, our results suggest that fungicides could be used as an alternative additive to control WBPH.

## Figures and Tables

**Table 1:** Primers and probes used in the polymerase chain reaction (PCR), nested PCR, denaturing gradient gel electrophoresis (DGGE), and quantitative PCR analyses.

**First-round PCR**	
NS5	5′-AACTTAAAGGAATTGACGGAAG-3′
ITS4	5′-TCCTCCGCTTATTGATATGC-3′
**Second round PCR**	
GCclampITS1	5′-CGCCCGGGGCGCGCCCCGGGCGGGGCGGGGGCAC GGGGGGCCGTAGGTGAACCTGCGG-3′
ITS2	5′-GCTGCGTTCTTCATCGATGC-3′
GCclampITS3	5′-CGCCCGGGGCGCGCCCCGGGCGGGGCGGGGGCAC GGGGGGCATCGATGAAGAACGCAGC-3′
ITS4	5′-TCCTCCGCTTATTGATATGC-3′
**Absolute quantitative real-time PCR**	
*Ascomycetes* symbiotes 1f(as1f)	5′-CACCCGAGGGGTCGAGGTGA-3′
*Ascomycetes* symbiotes 1r(as1r)	5′-GCAGCGAAATGCGATAAGTAATGTGAAT-3′
*Cladosporium halotolerans* 1f (ch1f)	5′-GCACCCTTTAGCGAATAGTT-3′
*Cladosporium halotolerans* 1r (ch1r)	5′-CGAGCGTCATTTCACCAC-3′

f: forward primer; r: reverse primer.

**Table 2:** BLAST and sequence analysis of selected DGGE bands.

No.	Length	Closest Related Species	Ident.	GenBank Accession No.	Primers
1	283	*Alternaria alternata* isolate Alt-C71	100.00%	MN044804.1	a
2	274	*Periconia macrospinosa* isolate A10E	100.00%	JQ781723.1	a
3	244	*Alternaria alternata* strain PB-56	100.00%	MK333976.1	a
4	302	Uncultured Alternaria clone FUN55	100.00%	KC920881.1	a
5	338	*Fusarium* sp. strain FSP	100.00%	MN200310.1	b
6	337	*Cladosporium halotolerans* FCG 1829	100.00%	LC414361.1	b
7	407	*Naganishia albida* isolate KDLYL12-1	100.00%	JX174413.1	b
8	469	Uncultured fungus clone S44T_39	99.15%	KU164594.1	b
9	463	Uncultured marine fungus clone S2D3-21	99.35%	JX269268.1	b
10	461	Uncultured fungus clone	99.57%	MF510813.1	b
11	273	Uncultured fungus clone ZSH201205-35	100.00%	KX515492.1	b
12	438	*Sogatella furcifera* yeast-like symbiont (*Ascomycetes* symbiotes) 18S-ITS	92.05%	JF732896.1	b

a: Amplified using primers NS5–ITS4 and GCclampITS1-ITS2; b: Amplified using primers NS5–ITS4 and GCclampITS3-ITS4.

**Table 3:** Effect of tested fungicides on yeast-like symbionts (YLS_ abundance and adult female white-backed planthopper (WBPH) survival rate.

Fungicide	Dose/L	Day 1			Day 3			Day 5		
		Mean ± SE YLS Count (×10^4^/Insect)	Ratio Index (%)	Survival (%)	Mean ± SE YLS Count (×10^4^/Insect)	Ratio Index (%)	Survival (%)	Mean ± SE YLS Count (×10^4^/Insect)	Ratio Index (%)	Survival (%)
Water	-	17.43 ± 0.603 ^a^	100.0	100.0	18.31 ± 1.332 ^a^	100.0	97.8	21.97 ± 1.562 ^a^	100.0	95.6
PR	4.28 g	9.83 ± 1.527 ^c^	56.4	95.6	10.88 ± 0.601 ^b^	59.4	88.9	5.29 ± 0.317 ^b^	24.1	71.1
PH	2.00 mL	14.08 ± 1.010 ^b^	80.8	93.3	10.20 ± 0.198 ^bc^	55.7	86.7	5.08 ± 1.627 ^b^	23.2	57.8
TT	0.30 g	8.46 ± 0.301 ^c^	48.6	91.1	8.83 ± 0.654 ^c^	48.2	75.6	4.16 ± 1.816 ^bc^	19.1	51.1
PY	1.88 mL	16.08 ± 0.144 ^a^^b^	92.3	95.6	4.00 ± 0.500 ^d^	21.8	77.8	2.75 ± 0.433 ^c^	12.7	48.9
IP	2.00 mL	4.16 ± 0.144 ^d^	23.9	93.3	2.91 ± 1.100 ^d^	15.9	71.1	2.02 ± 0.202 ^c^	9.2	42.2

^a–d^: Different letters denote significant differences in values among treatments in the same column and same day (*p* < 0.05).

## References

[1] Nagadhara D., Ramesh S., Pasalu I.C., Rao Y.K., Sarma N.P., Reddy V.D., Rao K.V. (2004). Transgenic rice plants expressing the snowdrop lectin gene (gna) exhibit high-level resistance to the whitebacked planthopper (*Sogatella furcifera*). Theor. Appl. Genet..

[2] Miao Y.T., Deng Y., Jia H.K., Liu Y.D., Hou M.L. (2018). Proteomic analysis of watery saliva secreted by white-backed planthopper, *Sogatella furcifera*. PLoS ONE.

[3] Nasu S. (1963). Studies on some leafhoppers and planthoppers which transmit virus diseases of rice plant in Japan. Bull. Kynshu Agric. Exp. Stn..

[4] Gomez-Polo P., Ballinger M.J., Lalzar M., Malik A., Ben-Dov Y., Mozes-Daube N., Perlman S.J., Iasur-Kruh L., Chiel E. (2017). An exceptional family: Ophiocordyceps-allied fungus dominates the microbiome of soft scale insects (Hemiptera: Sternorrhyncha: Coccidae). Mol. Ecol..

[5] Kobiałka M., Michalik A., Walczak M., Szklarzewicz T. (2018). Dual “bacterial-fungal” symbiosis in deltocephalinae leafhoppers (Insecta, Hemiptera, Cicadomorpha: Cicadellidae). Microb. Ecol..

[6] Matsuura Y., Moriyama M., Łukasik P., Vanderpool D., Tanahashi M., Meng X.-Y., McCutcheon J.P., Fukatsu T. (2018). Recurrent symbiont recruitment from fungal parasites in cicadas. Proc. Natl. Acad. Sci. USA.

[7] Hou Y., Ma Z., Dong S.Z., Chen Y.H., Yu X.P. (2013). Analysis of yeast-like symbiote diversity in the brown planthopper (BPH), *Nilaparvata lugens* Stål, using a novel nested PCR-DGGE protocol. Curr. Microbiol..

[8] Dong S.Z., Pang K., Bai X., Yu X.P., Hao P.Y. (2011). Identification of two species of yeast-like symbiotes in the brown planthopper, *Nilaparvata lugens*. Curr. Microbiol..

[9] Cao W., Ma Z., Chen Y.H., Yu X.P. (2015). *Pichia anomala*, a new species of yeast-like endosymbionts and its variation in small brown planthopper (*Laodelphax striatellus*). J. Biosci. Bioeng..

[10] Xu H., Zheng X., Tong Z., Lu Z., Chen J., Yu X., Tao L. (2000). Effects of insecticides on the symbiotes in brown planthopper. Acta Agric. Zhejiangensis.

[11] Zhang X., Yu X., Chen J. (2008). High Temperature Effects on Yeast-like Endosymbiotes and Pesticide Resistance of the Small Brown Planthopper, *Laodelphax striatellus*. Rice Sci..

[12] Nasu S., Kusumi T., Suwa Y., Kita H. (1981). Symbiotes of planthoppers: II. isolation of intracellular symbiotic microorganisms from the brown planthopper, *Nilaparata lugens* Stål, and immunological comparison of the symbiotes associated with rice planthoppers (Hemiptera: Delphacidae). Appl. Entomol. Zool..

[13] Noda H., Kawahara N. (1995). Electrophoretic karyotype of intracellular yeast-like symbiotes in rice planthoppers and anobiid beetles. J. Invertebr. Pathol..

[14] Noda H., Nakashima N., Koizumi M. (1995). Phylogenetic position of yeast-like symbiotes of rice planthoppers based on partial 18S rDNA sequences. Insect Biochem. Mol. Biol..

[15] Suh S.O., Noda H., Blackwell M. (2001). Insect symbiosis: Derivation of yeast-like endosymbionts within an entomopathogenic filamentous lineage. Mol. Biol. Evol..

[16] Yu H., Ji R., Ye W., Chen H., Lai W., Fu Q., Lou Y. (2014). Transcriptome analysis of fat bodies from two brown planthopper (*Nilaparvata lugens*) populations with different virulence levels in rice. PLoS ONE.

[17] Xue J., Zhou X., Zhang C.X., Yu L.L., Fan H.W., Wang Z., Xu H.J., Xi Y., Zhu Z.R., Zhou W.W. (2014). Genomes of the rice pest brown planthopper and its endosymbionts reveal complex complementary contributions for host adaptation. Genome Biol..

[18] Noda H., Koizumi Y. (2003). Sterol biosynthesis by symbiotes: Cytochrome P450 sterol C-22 desaturase genes from yeast-like symbiotes of rice planthoppers and anobiid beetles. Insect Biochem. Mol. Biol..

[19] Sasaki T., Kawamura M., Ishikawa H. (1996). Nitrogen recycling in the brown planthopper, Nilaparvata lugens: Involvement of yeast-like endosymbionts in uric acid metabolism. J. Insect Physiol..

[20] Bai X., Dong S.Z., Pang K., Bian Y.L., Yu X.P. (2010). Identification of one yeast-like symbiont from the small brown planthopper, *Laodelphax striatellus* (Fallén) (Homoptera: Delphacidae). Acta Entomol. Sin..

[21] Su J., Wang Z., Zhang K., Tian X., Yin Y., Zhao X., Shen A., Gao C.F. (2013). Status of Insecticide Resistance of the Whitebacked Planthopper, *Sogatella furcifera* (Hemiptera: Delphacidae). Fla. Entomol..

[22] Shentu X.P., Li D.T., Xu J.F., She L., Yu X.P. (2016). Effects of fungicides on the yeast-like symbiotes and their host, *Nilaparvata lugens* Stål (Hemiptera: Delphacidae). Pestic. Biochem. Physiol..

[23] Shentu X.P., Wang X.L., Xiao Y., Yu X.P. (2019). Effects of fungicide propiconazole on the yeast-like symbiotes in brown planthopper (BPH, *Nilaparvata lugens* Stål) and its role in controlling BPH infestation. Front. Physiol..

[24] Mukherjee I., Gopal M., Chatterjee S.C. (2003). Persistence and Effectiveness of Iprodione against Alternaria blight in Mustard. Bull. Environ. Contam. Toxicol..

[25] Smilanick J.L., Mansour M.F., Gabler F.M., Goodwine W.R. (2006). The effectiveness of pyrimethanil to inhibit germination of *Penicillium digitatum* and to control citrus green mold after harvest. Postharvest Biol. Technol..

[26] Pan L., Lai D. (2009). Ameliorative effects of Propineb WP on sheath blight and brown spot disease of rice. Guangxi Agric. Sci..

[27] Hu J., Hong C., Stromberg E.L., Moorman G.W. (2007). Effects of propamocarb hydrochloride on mycelial growth, sporulation, and infection by *Phytophthora nicotianae* isolates from Virginia nurseries. Plant Dis..

[28] Mohapatra S., Ahuja A.K., Deepa M., Jagadish G., Prakash G., Kumar S. (2010). Behaviour of trifloxystrobin and tebuconazole on grapes under semi-arid tropical climatic conditions. Pest Manage. Sci..

[29] Cao W., Zheng M.A., Xiao-Ping Y.U. (2015). Isolation and sensitivity to fungicides of the yeast-like symbiont Pichia anomala (Hemiascomycetes: Saccharomycetaceae) from Laodelphax striatellus (Hemiptera: Delphacidae). Acta Entomol. Sin..

[30] Doaré-Lebrun E., El Arbi A., Charlet M., Guérin L., Pernelle J.-J., Ogier J.-C., Bouix M. (2006). Analysis of fungal diversity of grapes by application of temporal temperature gradient gel electrophoresis–potentialities and limits of the method. J. Appl. Microbiol..

[31] Noda H., Omura T. (1992). Purification of yeast-like symbiotes of planthoppers. J. Invertebr. Pathol..

[32] Liang H.W., Wang C.Z., Li Z., Luo X., Zou G. (2008). Improvement of the silver-stained technique of polyacrylamide gel electrophoresis. Hereditas.

[33] Wheeler D.L., Barrett T., Benson D.A., Bryant S.H., Canese K., Chetvernin V., Church D.M., DiCuccio M., Edgar R., Federhen S. (2007). Database resources of the National Center for Biotechnology Information. Nucleic Acids Res..

[34] Chen C.C., Cheng L.L., Hou R.F. (1981). Studies on the intracellular yeast-like symbiote in the Brown Planthopper, *Nilaparvata lugens* Stål: II. Effects of antibiotics and elevated temperature on the symbiotes and their host. Z. Angew. Entomol..

[35] Pang K., Dong S.Z., Hou Y., Bian Y.L., Yang K., Yu X.P. (2012). Cultivation, identification and quantification of one species of yeast-like symbiotes, *Candida*, in the rice brown planthopper, *Nilaparvata lugens*. Insect Sci..

[36] Chang X.-N., Wei H., Xiao N.-W., Li J.-S., Han L., Chen F.-J. (2011). Effects of elevated CO_2_ and transgenic Bt rice on yeast-like endosymbiote and its host brown planthopper. J. Appl. Entomol..

[37] Ying H.L., Hou R.F. (1987). Physiological roles of a yeast-like symbiote in reproduction and embryonic development of the brown planthopper, *Nilaparvata lugens* Stål. J. Insect Physiol..

[38] Chen J.M., He Y.P., Zhang J.F., Na L., Chen L.Z., Yu X.P. (2009). Effects of insecticides and fungicides on growth of endosymbiotes isolated from the brown planthopper, *Nilaparvata lugens*. Plant Prot..

